# Treatment of Chordomas and Chondrosarcomas With CyberKnife Robotic Hypofractionated Radiosurgery: A Single Institution Experience

**DOI:** 10.7759/cureus.17012

**Published:** 2021-08-08

**Authors:** Morena Sallabanda, Rafael Garcia, Luis Lorenzana, Iciar Santaolalla, Javier Abarca, Kita Sallabanda

**Affiliations:** 1 Radiation Oncology, Genesis Care Cyberknife Center, Madrid, ESP; 2 Neurosurgery, Genesis Care Cyberknife Center, Madrid, ESP; 3 Medical Physics, Genesis Care Cyberknife Center, Madrid, ESP; 4 Neurological Surgery, Hospital General de Alicante, Madrid, ESP; 5 Radiosurgery/Neurosurgery, Hospital Clinico Universitario San Carlos, Madrid, ESP

**Keywords:** chondrosarcoma, chordoma, hypofractionation, radiosurgery, cyberknife

## Abstract

Introduction

The aim of this study is to determine the efficacy and safety of CyberKnife® (Accuray, Inc., Sunnyvale, CA) hypofractionated radiosurgery (HfRS) in the treatment of chordomas and chondrosarcomas.

Methods

A total of 24 patients retrospectively identified with chordomas (19 patients) or chondrosarcomas (five patients) were treated between 2012 and 2019 with HfRS as monotherapy or an adjuvant, rescue, or combination therapy. Tumors were located in the skull base (75%) and vertebral spine (25%). Of these, 19 patients underwent previous partial resection and four patients received previous conventional external beam radiation therapy (EBRT) (60-74 Gy). Exclusive or rescue HfRS (20 patients) was administered in five fractions with a median dose of 37.5 Gy (30-40 Gy). Combined tomotherapy-EBRT treatment (median dose: 54 Gy) and HfRS (16.5-30 Gy in 3-12 fractions) were performed in four patients with bulky chordomas.

Results

The median follow-up from HfRS was 28 months. During clinical follow-up, no deaths were registered with overall survival (OS) of 100% and the actuarial local recurrence-free survival (LRFS) was 93% at one year, 85% at three years, and 68% at five years. Acute toxicity related to HfRS was present in a single patient.

Conclusions

It is seen that HfRS is effective and safe for chordomas and chondrosarcomas, with rates of LRFS comparable to other radiation modalities.

## Introduction

Chordomas are rare, slow-growing tumors developing from remnants of the embryonic notochord in the intracranial clivus, sacrococcygeal region, and mobile spine [1,2]. Despite the low rate of metastases, these tumors present local invasiveness and an extremely high local recurrence rate. Similarly, chondrosarcomas have slow growth potential with a tendency to recur locally. They commonly arise in the skull base or vertebral spine from primitive mesenchymal or cartilaginous matrix cells [3,4]. Although chordomas are considered clinically more aggressive than chondrosarcomas, their propensity for particular anatomical locations and high risk of local recurrence has conditioned a similar therapeutic approach [5-7].

The standard dictates maximal safe surgical resection; nonetheless, radical removal is feasible in less than 50% of the cases, associated with considerable postoperative morbidity and mortality due to the invasion of critical structures [8,9]. Thus, optimizing the efficacy of radiation therapy represents a critical step in improving patient outcomes. Given their tendency for local recurrence, chordomas are classically considered radioresistant, prompting the prescription of large doses in the range of 60-75 Gy over a period of five to seven weeks. The adverse effects associated with such doses using conventional fractionated photon therapy, in turn, motivated the application of charged particles such as proton beam radiation therapy (PBRT) [6,10,11].

The α/β ratio represents a measure of the sensitivity of a tumor to varying dose-per-fraction regimens. Tumors with a low ratio (<4 Gy) will be more sensitive to the effects of high-dose single sessions [12]. After the examination of historical studies and institutional experience, Georgetown University suggested an α/β ratio value of 2.45 Gy for chordomas, and the same ratio was assumed for chondrosarcomas. Thus, the estimation of a low α/β ratio in chordomas and chondrosarcomas predicts improved outcomes with large doses in hypofractionation regimen [13].

We present a series of 24 patients with chordoma or chondrosarcoma from a single institution treated with CyberKnife® (Accuray, Inc., Sunnyvale, CA) hypofractionated radiosurgery (HfRS). In the present study, overall survival (OS), local recurrence-free survival (LRFS), and toxicity profiles are analyzed with the aim of presenting results comparable to those of PBRT and contributing to the increase in clinical evidence of HfRS treatment efficacy in these tumors.

## Materials and methods

A total of 24 patients (14 males and 10 females) with a median age at diagnosis of 52 years (range: 6-85 years) and biopsy-proven chordomas (n=19, 79%) or chondrosarcomas (n=5, 21%) were treated between 2012 and 2019 with CyberKnife robotic HfRS. Treatment modalities were classified as adjuvant, monotherapy, in combination with external beam radiation therapy (EBRT), or rescue treatment after a first local failure. These patients were retrospectively identified and demographic, clinical, and therapeutic data were extracted from the institutional registry and medical records. All patients included in the study, or their legal guardians, signed an informed consent agreeing to receive the proposed treatment.

Patients were staged with body CT and MRI scans at diagnosis and no distant metastases were present. The tumors were situated in the clivus (75%), cervical spine (12.5%), and lumbosacral spine (12.5%). Symptoms varied according to tumor location, with cranial nerve sequelae (58%) in skull base lesions, motor (21%), and/or sensitive (13%) dysfunction in the mobile spine and sacral tumors. Pain was present in 50% of patients. of the patients, 19 (79%) underwent surgical resection but none reported as gross total resection. Patient selection for surgery was based on tumor extent and proximity to critical structures. In addition, four patients received previous EBRT (60-74 Gy). Exclusive, adjuvant, or rescue CyberKnife HfRS treatments (20 patients) were all administered in five consecutive or alternate fractions with a median dose of 37.5 Gy in chordomas (range: 30-40 Gy) and 30 Gy in chondrosarcomas (range: 30-35 Gy). A minimum prescription dose of 30 Gy in five fractions was aimed in exclusive HfRS regimens. Patients who could not benefit from exclusive CyberKnife HfRS received combined treatment with tomotherapy EBRT (four patients). All of these patients had bulky chordomas with large radiation treatment fields. The median dose administered with tomotherapy was 54 Gy (range: 50-60 Gy), with conventional fractionation. Subsequently, they received a CyberKnife boost that allowed a safer dose escalation, administering 16.5 Gy in three sessions, 24 Gy in 12 sessions, 20 Gy in five sessions, and 30 Gy in five sessions.

Patients were followed at three, six, and 12 months and, thereafter, at six-month intervals. The median follow-up from HfRS was 28 months (6-72 months). Evaluation included MRI, information on interval history, and neurological examination. Lesions were considered nonrecurrent if radiographic studies indicated no evidence of local or distant disease progression and the patient had clinically improved or was unchanged. In case of the appearance of new symptoms, a differential diagnosis between relapse and treatment toxicity was made using additional imaging as positron emission tomography (PET) scan. Relapse was classified as in-field or out-of-field.

All patients underwent simulation CT and MRI (1.25 mm slice thickness) scans in a supine position. For intracranial or cervical spine tumors, patients were immobilized with a thermoplastic mask. For target delineation, CT, MRI, and, in some cases, PET-CT images were fused. The gross tumor volume (GTV) and critical structures were delineated by the treating radiation oncologist and neurosurgeon. The clinical tumor volume (CTV) was equivalent to the GTV when definitive HfRS was performed, except in the cases of spine tumors, in which the CTV was expanded to include the entire vertebral body. For patients receiving postoperative HfRS, the CTV included any residual gross disease and regions at risk for local recurrence, such as bone or soft tissue. When a combined treatment with EBRT was performed, HfRS was delivered as a boost to the GTV. A planning tumor volume (PTV) of 1-2 mm was generated in exceptional occasions when metal artifacts from surgery or difficulties in image fusion reduced the accuracy in contouring or setup.

The CyberKnife is a robotic, image-guided, stereotactic radiosurgery system, with a 6-MV linear accelerator mounted on a fully articulated robotic arm capable of translational and rotational movements to target tumors without rigid external fixation. Real-time image guidance was accomplished by monitoring patient position every 30-60 seconds. Translations and rotations were aligned during patient setup and automatically corrected during treatment delivery [13-14]. 

**Figure 1:**
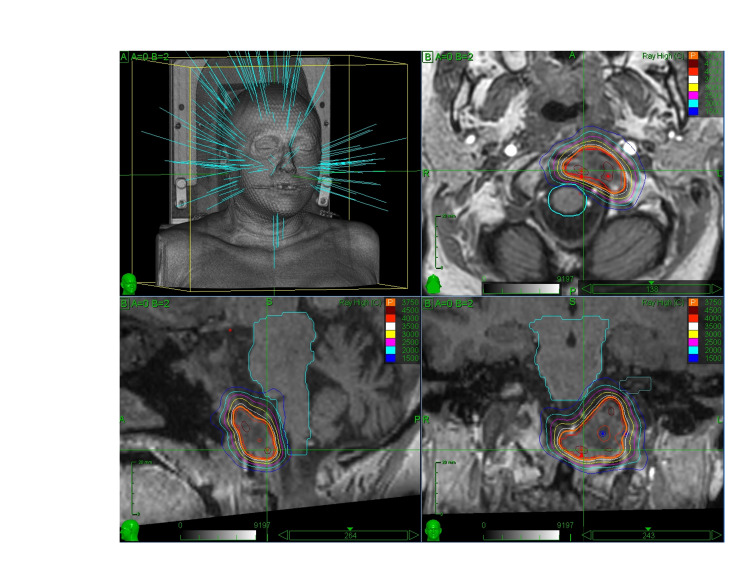
CyberKnife MultiPlan® (Accuray, Inc., Sunnyvale, CA) planning of a clival chordoma treatment with 37.5 Gy in five fractions.

Dose-volume histograms were calculated for target lesions and all critical structures. The conformity index, homogeneity index, and coverage were used to evaluate radiation dosimetry. When necessary, PTV or CTV coverage with the prescription dose was sacrificed to respect normal tissue tolerance. A maximum punctual dose (0.1cc) of 30 Gy, 31 Gy, and 32 Gy were permitted in the spinal cord, brainstem, and cauda equina, respectively, for five fraction regimens. In Figure 1, a CyberKnife HfRS plan of a clival chordoma treated with 37.5 Gy in five fractions is shown.

For the descriptive analysis, frequency tables were created on qualitative variables. In quantitative variables, mean, range, and median were described. For the analysis of survival, the date of diagnosis and HfRS treatment to the date of appearance of the corresponding event or the date of the last follow-up was collected. The LRFS was estimated using the Kaplan-Meier method. In the univariate analysis, the log-rank test was used to compare survival curves. In all the analyses, the level of statistical significance was placed in a type I error of less than 5% (P<0.05). The confidence interval (CI) was designated 95%.

## Results

Baseline characteristics of the 24 patients with single lesions of chordoma or chondrosarcoma and HfRS or combined treatment characteristics are listed in Tables 1 and Table 2, respectively.

**Table 1:** Clinical characteristics in chordoma and chondrosarcoma patients from hypofractionated radiosurgery EBRT: External beam radiation therapy; PBRT: Proton beam radiation therapy; Gy: Gray

Parameter	Results
Age at diagnosis (years)	52 (6–85)
Male sex	14 (58%)
Female sex	10 (42%)
Histological type: Chordoma, Chondrosarcoma grade I-II	19 (79%), 5 (21%)
Tumor locations: Clivus, Cervical spine, Lumbosacral spine	18 (75%), 3 (12.5%), 3 (12.5%)
Symptoms at diagnosis: Pain, Cranial nerve deficit, Motor dysfunction, Sensitive dysfunction	12 (50%), 14 (58%), 5 (21%), 3 (13%)
Surgical resection: Chordoma, Chondrosarcoma	17 (89.5%), 2 (40%)
Type of surgery: Gross total resection, Subtotal resection, No resection (biopsy)	0 (0%), 19 (79%), 5 (21%)
Postoperative symptoms: Improved or unchanged, Pain, Cranial nerve deficit, Motor dysfunction, Sensitive dysfunction	10 (42%), 2 (8%), 5 (21%), 6 (25%), 1 (4%)
Previous EBRT: PBRT (74 Gy), Tomotherapy EBRT (60-74 Gy)	1 (4%), 3 (12.5%)

**Table 2:** Hypofractionated radiosurgery and combined treatment characteristics in chordoma and chondrosarcoma patients. HfRS: Hypofractionated radiosurgery; BED: Biologically effective dose; Gy: Gray; EBRT: External beam radiation therapy;

Parameter	Results
Postoperative HfRS: Median dose (Gy)/ fractions, Median BED (Gy)	37.5/5 (30–40/5), 152.3 (103.5–170.6)
Chordoma reirradiation HfRS: Median dose (Gy)/ fractions, BED (Gy)	35/5 (30-37.5/5), 135 (103.5–152.3)
Chordoma combined HfRS + EBRT: Median dose (Gy)/ fractions EBRT, Dose (Gy)/ fractions HfRS, Global BED (Gy)	54/27 (50–60/25–30), 16.5/3–20/5–30/5–24/12, 156,7 (134,4–205,2)
Chordoma exclusive HfRS: Median dose (Gy)/ fractions, BED (Gy)	37.5/5 (35–40/5), 152.3 (135–170.6)
Chondrosarcoma: Median dose (Gy)/ fractions, BED (Gy)	30/5 (30–35/5), 103.5 (103.5–135)
CTV/PTV: in cc CTV, in cc for combined HfRS + EBRT	8.7 (1.8–59), 37.7 (11.9–59)
Coverage %	98 (80–100)
Conformity index	1.19 (1.11–2)
Homogeneity index	1.22 (1.14–1.37)

Analysis of survival and local control

**Figure 2:**
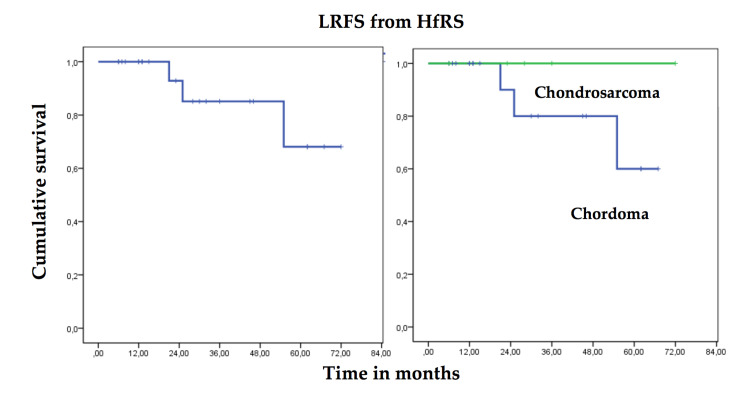
Global actuarial LRFS in chordomas and chondrosarcomas from HfRS and comparison of actuarial LRFS curves between chordomas and chondrosarcomas LRFS: Local recurrence-free survival; HfRS: Hypofractionated radiosurgery

At the time of the last clinical follow-up, all the patients included in the series were alive (OS 100%). Median follow-up from diagnosis was 55 months (range: 8-173 months). Patients were followed after HfRS for a median of 28 months (range: 6-72 months). The global actuarial LRFS was 93% at one year (95% CI 86, 1-99,9%), 85% at three years (95% CI 75, 3-94,7%) and 68% at five years (95% CI 51-85%). The actuarial LRFS for chordoma patients was 90% at one year (95% CI 80,5-99,5%), 80% at three years (95% CI 67, 4-92,6%), and 60% at five years (95% CI 40, 3-79,7%), while chondrosarcoma patients did not relapse locally during follow-up. Of the 19 chordoma patients, three (16%) experienced recurrence at a mean of 34 months after HfRS (range: 21-55 months). All relapses were locoregional, but out of the radiation field, probably related to the surgical approach and all patients could be rescued with either surgery or EBRT. Two of the patients, who had local recurrence, received combined treatment with tomotherapy EBRT and CyberKnife HfRS. None of the patients included in this series was diagnosed with metastatic disease during follow-up. The global Kaplan-Meier LRFS curve from HfRS and comparison of actuarial LRFS curves in chordomas and chondrosarcomas are shown in Figure 2.

Univariate analysis

**Figure 3:**
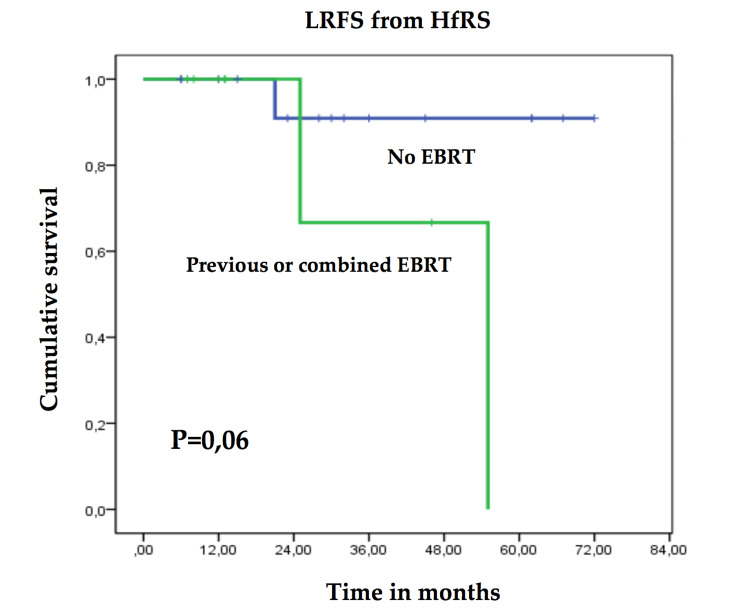
Univariate analysis comparing actuarial LRFS curves between previous or combined vs. absence of EBRT in chordoma and chondrosarcoma patients LRFS: Local relapse-free survival; EBRT: External beam radiation therapy; HfRS: hypofractionated radiosurgery

In the univariate analysis, the comparison of actuarial LRFS curves showed that despite the differences in the relapse pattern between chordomas and chondrosarcomas, statistical significance was not reached. Tumor location (skull base versus spine) did not demonstrate a significantly different response pattern. Clinical characteristics such as age, gender, or tumor volume did not correlate with local control either. Although the type of surgery had no impact on LRFS (none of the patients included in the series could benefit from a gross total resection), previous or combined EBRT showed a trend towards statistical significance (P=0.06) as a poor prognostic factor for local relapse (Figure 3). In terms of exclusive HfRS in chordomas, prescription dose, BED, and CTV or PTV coverage had no statistical impact in local control.

Treatment-associated toxicity

Severe acute toxicity related to HfRS was present in a single patient. This patient had initially presented with a clival chordoma, diagnosed in August 2014 due to lower cranial nerve involvement. The initial treatment consisted of partial resection and tomotherapy EBRT with a prescription dose of 74 Gy in 37 fractions. The patient recovered most of the cranial nerve damage and presented no signs of relapse until October 2019, when symptoms of headache, altered speech, and left tongue atrophy appeared. MRI showed an 8cc left clival lesion that was treated with partial resection. A residual CTV of 5.2cc was re-irradiated with CyberKnife HfRS prescribing 37.5 Gy in five fractions to the margin. Within three months after HfRS, the patient was diagnosed with a clival fistula and meningitis. These complications resolved with non-invasive medical treatment and there was no evidence of late toxicity other than an occasional headache, which was present at the last follow-up, with no sign of local relapse.

There were no disabling acute or late toxicities in the remaining patients included in the series. For the most part, neurological deficits were already present at diagnosis or after surgical resection as exposed in Table 1. After HfRS these symptoms improved or remained unchanged.

## Discussion

In the present study, a series of 24 patients with chordomas or chondrosarcomas treated with HfRS is reported. Despite a number of limitations, which include two different tumor histologies, the small number of patients, low incidence of reported events, relatively short follow-up period, and retrospective nature, this series represent one of the largest cohorts of chordoma and chondrosarcoma patients treated with CyberKnife HfRS presented in literature, to our knowledge. The difficulty in recruiting patients is related to the low prevalence of these tumors and the fact that HfRS is not considered standard in the treatment course of chordomas and chondrosarcomas. Thus, the fundamental relevance of publishing this series lies in the contribution of data to the global literature, which is scarce, allowing further investigations and possibilities for pooled analysis.

The management of chordomas and chondrosarcomas remains challenging. Surgical margin is the most important determinant of survival, and most patients who are treatable with wide margins survive, but the optimal treatment of these tumors with gross total resection is infrequent because of neurovascular involvement, so recurrence with surgery alone is a significant risk [15-18]. In this series, gross total resection could not be completed in any patient and thus surgery did not impact as a positive prognostic factor for LRFS.

In this regard, adjuvant EBRT after resection has been used historically with median doses of 60 to 66.6 Gy resulting in five-year local control rates ranging from 23-50% [15-19]. Technological advances in the field, such as high-dose image-guided intensity-modulated radiotherapy, have allowed increased precision permitting higher doses for chordomas up to 76 Gy (BED 138 Gy) and for chondrosarcomas up to 70 Gy (BED 127 Gy), and this has further pushed these benefits for five-year local control rates of 65% and 88%, respectively [15,20]. Though promising, the use of high-dose photons is limited by the lower ability to spare nearby critical organs and the need for daily CT scans over a total of 40 fractions. Therefore, particle therapy has risen to prominence because of its higher potential conformality and unique dose deposition. The principal studies in PBRT for these tumors appear to show more favorable outcomes in comparison with traditional photons [15,21]. In a study at Loma Linda University Medical Center using a proton dose of 65 to 79 Gy, five-year local control and OS rates were 59% and 79%, respectively, for chordomas and 75% and 100%, respectively, for chondrosarcomas in the base of the skull. This trend has been supported in proton studies for chordomas and chondrosarcomas of the spine, which have shown potential benefits of dose escalation to 72-77.4 Gy with improved five-year local control rates in comparison with photon techniques. High-dose (>70 Gy, BED >127 Gy) PBRT provides 72-80% OS and 60-81% local control at five years with low toxicity and sparing of critical structures. However, the limited availability of these facilities along with their long treatment course, often seven weeks, is trying for patients and remains an obstacle to such an approach being widely adopted as a standard of care [7,15,22-25]. Thus, the importance of exploiting HfRS therapy as a treatment modality has become increasingly apparent. 

Multiple studies have assessed single-fraction stereotactic radiosurgery (SRS) for chordoma and chondrosarcoma management mainly in skull-base location and demonstrated comparable results to PBRT [7,26-29]. The cited studies included series of 22-71 patients treated with SRS median doses ranging from 12.7-24 Gy (BED 78,5-259 Gy), with a median follow-up of 28-75 months. Five-year local control rates varied between 21-85%, depending on prescription doses (higher doses, ≥15 Gy, related with improved LRFS). Nevertheless, target volumes >7cc are significantly associated with worse tumor control. Symptomatic adverse radiation effects occurred in up to 10% of patients.

The steep dose gradient permitted by SRS and HfRS can similarly maximize dose radiation to the tumor while minimizing radiation to surrounding critical structures. Nonetheless, HfRS has theoretical advantages compared to single fraction treatment including lower risk of radiation toxicity in nearby critical structures and the possibility of safely treating larger tumor volumes with multiple fractions. A major advantage of the CyberKnife system is the ability to treat intracranial and extracranial lesions with complex shapes or locations that are difficult or impossible to treat using frame-based systems [7,30].

A limited number of published series evaluate CyberKnife HfRS for chordomas and chondrosarcomas [4,7,9,13,30]. The number of cases included in these series ranged from nine to 20 patients. Median follow-up from HfRS was 24-46 months. The majority of patients were treated in five fractions with a prescribed dose of 24-43 Gy (BED 52,7-194 Gy), depending on histology and the exclusive, adjuvant, or reirradiation context. Jiang et al. reported a five-year local control of 41% in chondrosarcomas and a three-year local control of 55% in chordomas. Vasudevan et al. achieved a three-year LRFS of 89% and Gwak et al. a two-year local control of 90% for chordomas and chondrosarcomas. Henderson et al., with the largest median follow-up, described a five-year LRFS of 59% in chordomas. Significant complications appeared in 5% to 20% of cases, almost exclusively when patients had received previous irradiation. The small sample sizes particularly affected the ability to identify patients subgroups based on either clinical parameters or histological subtypes that are more likely to benefit from HfRS, but in almost all studies, patients treated in a recurrence setting exhibited a significantly higher risk for local failure.

In the present series, as mentioned in the results, HfRS prescription doses ranged between 30-40Gy in five fractions, depending on the histology, tumor volume, and previous radiation. Chondrosarcoma patients received lower doses, with a median BED of 103.5 Gy, achieving an excellent local control rate, with no evidence of local relapse during follow-up, despite the absence of surgery in 60% of cases. It is important to emphasize that all patients were classified as grade I-II, which implies a more benign profile, consistent with these results.

On the other hand, chordoma patients treated in postoperative or exclusive settings received the higher prescription doses with a median BED of 152.3 Gy, whereas in the reirradiation context, median BED lightly descended to 135 Gy. The four patients with bulky chordomas who received combined treatment with tomotherapy EBRT and CyberKnife HfRS used different regimens to achieve a median BED of 156.7 Gy. It is important to emphasize that, despite the heterogeneity of the regimens used in this last subgroup of patients, these data show that when HfRS cannot be used as an exclusive treatment because of tumor extent, there is a role for HfRS as a boost for escalating dose more precisely and safely when EBRT alone is not capable of reaching therapeutic doses.

The global actuarial LRFS was 68% at five years and the actuarial LRFS for chordoma patients was 60% at five years, which, in an indirect comparison of the range of results obtained with PBRT, means non-inferiority. Due to the low rate of local relapses in chordoma patients (only three patients) during follow-up, statistical significance among prognostic factors was not achieved. The only trend towards statistical significance as a poor prognostic factor for local relapse was previous or combined EBRT, which could be explained because of the lower HfRS doses and bigger tumor volumes, though these factors did not associate for themselves as significant factors for local relapse. It is relevant to highlight that all relapses occurred locoregionally but out of the radiation field. This fact could be related to the surgical approach, which implies an excellent local control within the radiation field with the prescribed doses. The absence of a wider treatment margin (CTV or PTV) did not associate with higher local recurrence compared to other series and benefited the optimization of dosimetric parameters. Tumors located in the base of the skull compared with the vertebral spine had a similar response to multimodality treatment or exclusive HfRS treatment, suggesting that the radiosurgical approach should not vary despite the location.

Finally, in terms of toxicity, only one case, in the context of reirradiation, presented acute complications that were resolved at the time of the last follow-up. Although longer follow-up periods are required, this fact supposes the assumption of a favorable toxicity profile for this treatment modality.

## Conclusions

CyberKnife robotic HfRS for chordomas and chondrosarcomas is an effective treatment modality in different settings such as adjuvant, monotherapy, combination with EBRT, and rescue, with potential for dose escalation, precise targeting, steep dose reduction outside target volumes, and shorter treatment courses. This technique provides rates of LRFS comparable to published data for other advanced radiation modalities, with low toxicity. Prospective randomized data with larger sample sizes and longer follow-up periods should further validate this technique for patients with chordomas and chondrosarcomas.
